# Transcriptome Analysis Reveals the Immunoregulatory Activity of Rice Seed-Derived Peptide PEP1 on Dendritic Cells

**DOI:** 10.3390/molecules28135224

**Published:** 2023-07-05

**Authors:** Tingmin Qu, Shuwen He, Ying Wu, Yingying Wang, Ce Ni, Shiyu Wen, Bo Cui, Yunhui Cheng, Li Wen

**Affiliations:** 1 School of Food Science and Bioengineering, Hunan Provincial Key Laboratory of Cytochemistry, Changsha University of Science & Technology, Changsha 410114, China; treatmi@163.com (T.Q.); hsw1122108@163.com (S.H.); cyh@csust.edu.cn (Y.C.); 2 School of Food Science and Engineering, Qilu University of Technology, Jinan 250353, China

**Keywords:** transcriptome, rice-derived peptide, immunopeptide, dc2.4 cells, immunoregulatory mechanism

## Abstract

Some food-derived bioactive peptides exhibit prominent immunoregulatory activity. We previously demonstrated that the rice-derived PEP1 peptide, GIAASPFLQSAAFQLR, has strong immunological activity. However, the mechanism of this action is still unclear. In the present study, full-length transcripts of mouse dendritic cells (DC2.4) treated with PEP1 were sequenced using the PacBio sequencing platform, and the transcriptomes were compared via RNA sequencing (RNA-Seq). The characteristic markers of mature DCs, the cluster of differentiation CD86, and the major histocompatibility complex (MHC-II), were significantly upregulated after the PEP1 treatment. The molecular docking suggested that hydrogen bonding and electrostatic interactions played important roles in the binding between PEP1, MHC-II, and the T-cell receptor (TCR). In addition, the PEP1 peptide increased the release of anti-inflammatory factors (interleukin-4 and interleukin-10) and decreased the release of pro-inflammatory factors (interleukin-6 and tumor necrosis factor-α). Furthermore, the RNA-seq results showed the expression of genes involved in several signaling pathways, such as the NF-κB, MAPK, JAK-STAT, and TGF-β pathways, were regulated by the PEP1 treatment, and the changes confirmed the immunomodulatory effect of PEP1 on DC2.4 cells. This findings revealed that the PEP1 peptide, derived from the byproduct of rice processing, is a potential natural immunoregulatory alternative for the treatment of inflammation.

## 1. Introduction

With the increase in public health awareness, the adverse effects of unhealthy lifestyles on human immune function have sparked great concern. Immune function is a key means of preventing and controlling infections and tumors. In order to enhance regulation of immune function, the development of non-toxic and easily absorbed food-derived immunopotentiators has become a major research focus in recent years. Cereal-based food proteins undergo enzymatic hydrolysis by a range of enzymes, such as trypsin, pepsin, and alkaline protease, and may produce large numbers of bioactive peptides [1,2]. These peptides exhibit various beneficial functions, including antibacterial, antioxidative, anti-inflammatory, and even immunostimulatory activities [3,4,5].

Immunopeptides are short peptides that regulate immune activity and participate in signal transduction in immune cells, including dendritic cell (DC), which are the most important immune cell type [6]. Dendritic cells are the most efficient antigen-specific-presenting cells in the body. Through the exogenous antigen-processing-and-presentation pathway, exogenous peptides form a complex with major histocompatibility complex II (MHC-II) in DCs are exposed on the cell surfaces, and can be presented to CD4+ T-cells via the T-cell receptor (TCR), eventually encouraging CD4+ T-cells to participate in the immune response [7]. Immunopeptides can stimulate DCs to change from immature to mature, while mature DCs can upregulate both surface MHC-II and co-stimulatory molecules, such as clusters of differentiation 80 (CD80) and 86 (CD86) [8]. In addition, DCs play a key role in the regulation of inflammatory and anti-inflammatory responses [9]. Given the importance of DCs, the immunomodulatory effects of food-derived bioactive peptides on DCs raise growing concerns.

Previous research confirmed that many immune-related signaling pathways, such as the JAK-STAT (Janus kinase/signal transducers and activators of transcription), MAPK (mitogen-activated protein kinase), and NF-κB (nuclear factor kappa light chain enhancer of activated B cells) pathways, are modulated by bio-functional peptides [10,11]. However, few comprehensive analyses have investigated how rice-derived bioactive peptides are involved in immunity-related signaling pathways. In RAW264.7 cells, we showed that the inhibition of the NF-κB signaling pathway was associated with anti-inflammatory activity, which suggested that immunopeptides exert anti-inflammatory effects by inhibiting the release of NO, IL-6, IL-1β, and TNF-α [12,13]. However, those studies were preliminary, and more data are needed to identify the key genes that are closely related to the regulation of the immune response. In addition, some immunopeptides can participate in antitumor effects in the body [14]. These peptides may provide new alternatives for the development of functional foods with anticancer or immunomodulatory activities.

In our previous study, the immunopeptide PEP1(GIAASPFLQSAAFQLR) was screened through the trypsin hydrolysis of rice protein using bioinformatics tools. The PEP1 peptide was derived from prolamin 14E [15]. The present study investigated the molecular mechanism of the effect of the immunopeptide PEP1 on antigen-presenting DC2.4 cells by measuring cell viability, phenotypical changes, and cytokine expression. Transcriptomic tools and molecular biology methods were used to provide the first explanation of the molecular mechanism of the PEP1 peptide by identifying key genes regulated by PEP1.

## 2. Results

### 2.1. Effects of PEP1 Peptide on DC2.4-Cell Viability and Maturation

**Figure 1:**
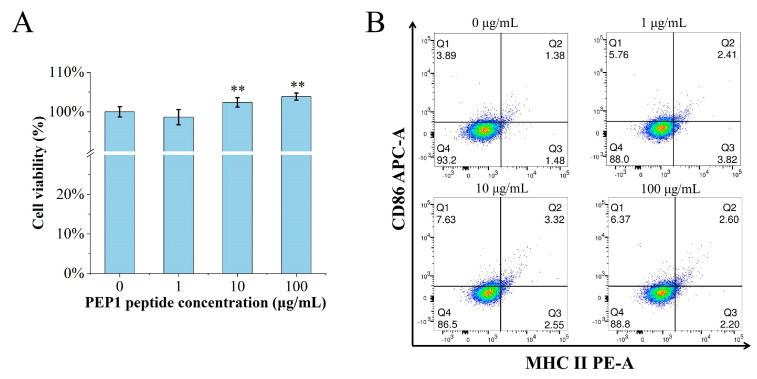
The effects of PEP1 on DC2.4 cells. (**A**) Cytotoxicity of different concentrations of PEP1 incubated with DC2.4 cells for 24 h and analyzed via the MTT assay. (**B**) CD86 and MHC-II expression was observed in DC2.4 cells treated with the PEP1 peptide. Q1: CD86 positive; Q2: double-positive of CD86 and MHC-II; Q3: MHC-II positive; Q4: double-negative of CD86 and MHC-II. ** *p* < 0.01 compared with the control group.

The MTT assay was performed to determine if or not PEP1 is toxic to DC2.4 cells. The cell viability was calculated for the groups of cells treated with different concentrations (0, 1, 10, and 100 μg/mL) of PEP1, named control, Pep1, Pep10, and Pep100, respectively. As shown in Figure 1A, there were no cytotoxic effects on the DC2.4 cells at the tested concentrations. Moreover, the in vitro DC2.4 cell viability assay indicated that the cells treated with 100 μg/mL of the PEP1 peptide had the highest viability (103.9% ± 4.3%).

**Figure 2:**
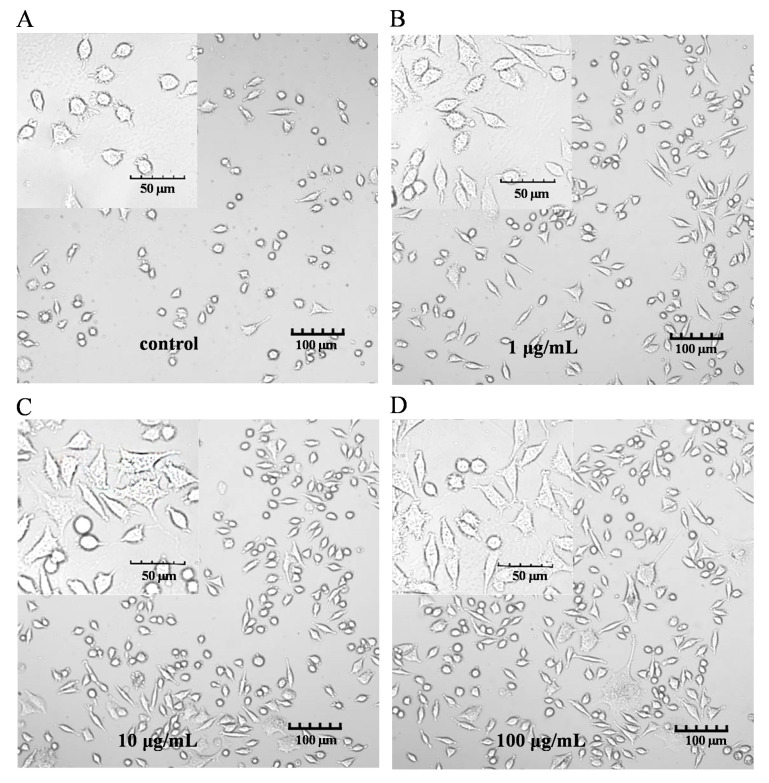
DC2.4 cell morphology. FV3000 Olympus laser confocal microscope was used to visualize the morphology (100× and 400×). (**A**) Control; (**B**) Pep1; (**C**) Pep10; (**D**) Pep100.

The characteristic markers of DCs, and CD86 and MHC-II molecules were used to evaluate DC maturation [16]. The expression of the CD86 and MHC-II molecules was found to be significantly upregulated in the PEP1-treated group, compared to that in the control group (Figure 1B). In addition, phenotypic changes in the DC2.4 cells were observed using a laser confocal microscope (Figure 2). The higher concentrations (10 and 100 μg/mL) of the PEP1 peptide clearly stimulated the proliferation of DC2.4 cells. Moreover, the cells exposed to 10 and 100 μg/mL of PEP1 for 24 h tended to aggregate, with the cytoplasm becoming comparable to those of the control and Pep1 groups. This indicated that 10 and 100 μg/mL of the PEP1 peptide also effectively stimulated the maturation of the DC2.4 cells [17].

### 2.2. Establishing a Full-Length Sequencing Database of the PEP1 Peptide-Stimulated DC2.4 Cells

**Figure 3:**
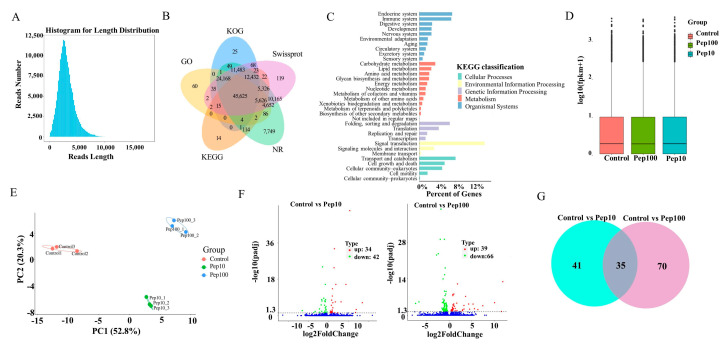
RNA-Seq analysis of gene expression in mice DC2.4 cells. (**A**) Length distribution of the full-length transcripts. (**B**) Summary of annotation in the five databases. (**C**) Kyoto Encyclopedia of Genes and Genomes (KEGG) annotation of the assembled full-length transcripts. (**D**) The transcript FPKM box plot. (**E**) Principal component analysis (PCA) diagram of the samples. (**F**) Volcano map of the differentially expressed genes compared to the control group. (**G**) Venn diagram of the differentially expressed genes compared to the control group.

Isoform sequencing (ISO-seq) technology was used to analyze the transcriptome of DC2.4 cells with and without the PEP1 peptide, and a total of 185.7 G subreads were generated (Table S2). After aligning to the reference sequences, clustering correction was conducted using IsoSeq3 polish. As a result, 351,412 circular consensus sequences (CCSs) were obtained, which had a total length of 1,039,560,299 bp and an average length of 2958.24 BP. LoRDEC software 0.9 was applied for further correction based on the RNA-seq data, and the CCSs obtained via IsoSeq3 clustering correction were then determined to be of a total length of 1,039,683,856 bp, with an average length of 2958.59 bp (Figure S2A). Finally, 138,482 transcripts were obtained by clustering the same isoforms and screening out the LnRNAs (Figure 3A). In total, 138,023 CDSs were predicted using ANGEL software, among which 1009 sequences were confident-complete, 55,849 were dumb-complete, 2989 were confident-5′partial, and 195 were confident-3′partial, while 68,744 were suspicious-NA with low confidence (Table S3). Subsequently, the transcripts were compared with the NR, GO, KEGG, Swiss-Prot, and KOG databases for functional annotation, in which 127,867 genes were annotated in total, with an annotation rate of 92.33%. Among them, 80,278, 69,205, 127,482, 119,762 and 93,928 sequences were annotated to the GO, KEGG, NR, Swiss-Prot, and KOG databases, respectively (Figure 3B and Table S4). Among the 119,762 genes, 62.86% were successfully annotated using non-redundant (nr) database subsets of *Mus musculus* (Figure S2B). Many genes were annotated by KOG as to the RNA processing and modification, followed by chromatin structure and dynamics, energy production and conversion, cell cycle control, cell division, and chromosome partitioning (Figure S2C). Gene ontology (GO) enrichment analysis showed that the genes were involved with 9 molecular functions, 21 biological processes, and 18 cellular component terms (Figure S2D). The highest percentage of the genes (15.5%) was annotated to signal transduction with KEGG environmental information possession. Over 20% of the successfully annotated genes were closely related to the immune response and signal transduction (Figure 3C).

The RNA-Seq reads were compared with the reference sequences generated by ISO-Seq sequencing. The average mapping rate of all libraries of RNA-Seq was 95.8%, suggesting that the PacBio library had a high degree of integrity (Table S5) [18]. The StringtTie software was used to calculate the expression of all genes as shown in Table S6. The FPKM box plots of the different groups were used to measure the differences among samples from the perspective of the overall dispersion of expression quantity (Figure 3D). PCA analysis of the duplicate samples of the three groups (control, Pep10, and Pep100) is presented in Figure 3E. The intra-group duplicates were clustered to each other and distant from the inter-group samples.

The differentially expressed genes (DEGs) among different experimental groups were analyzed as shown in Table S7. In total, the expression of 139 genes was changed in the PEP1 treatment groups to the control group. Compared with the control group, 34 and 39 genes were upregulated and down-regulated by 10 μg/mL and 100 μg/mL of the PEP1 peptide, respectively, as shown in the volcano mapping (Figure 3F). The expressions of 42 and 66 genes were regulated by 10 μg/mL and 100 μg/mL of the PEP1 peptide, respectively. The relationship of DEGs for each experimental group is shown in the Venn diagram, and the expression of 35 genes was found to be regulated by both 10 μg/mL and 100 μg/mL of the PEP1 peptide (Figure 3G). GO enrichment indicated that these 35 genes were involved in enzyme binding, kinase binding, transcription regulatory region DNA binding, etc. (Figure S3).

### 2.3. Screening of Immune-Related DEGs and Complicated Networks

**Figure 4:**
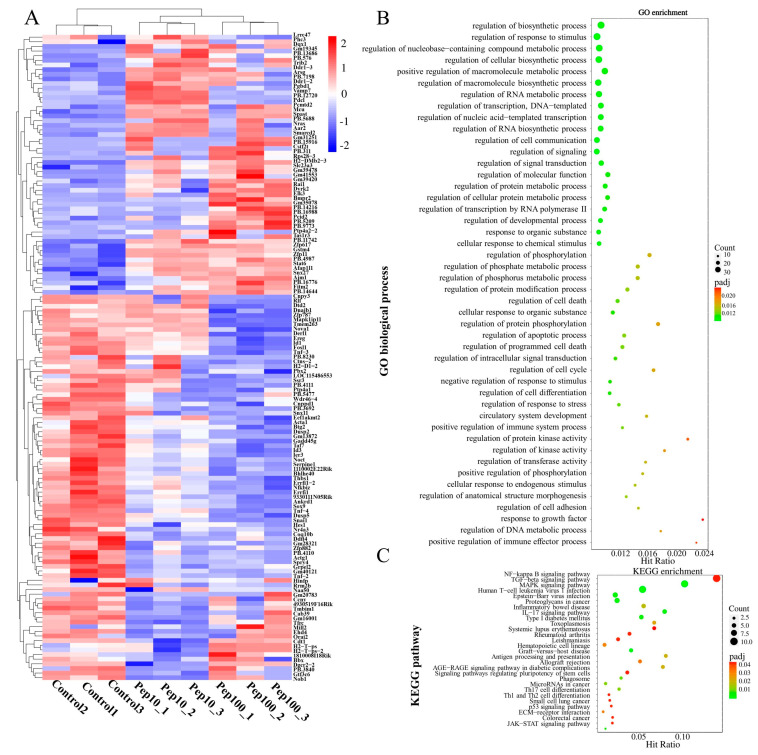
Identification of differentially expressed mRNAs after PEP1 treatment. (**A**) Heat map of gene differential expression of control, pep10 and pep100 groups. (**B**) Gene ontology (GO) classification of differentially expressed genes. (**C**) Kyoto Encyclopedia of Genes and Genomes (KEGG) annotation of differentially expressed genes.

To clarify the mechanism of action in PEP1-stimulated immune regulation, GO, KEGG enrichment, and protein–protein interactions (PPIs), analyses were performed on DEGs. Hierarchical clustering analysis of the up- or down-regulated genes of the treatment groups, compared with the control group, was revealed by the heat map (Figure 4A). The details of the expression FMPK values, GO, and KEGG enrichment of the DEGs are provided in Table S7. The highest GO enrichments of the 139 DEGs were related to the regulation of biosynthetic processes (GO:0009889), followed by the regulation of the response to the stimulus (GO:0048583) and regulation of nucleobase-containing compound metabolic processes (GO:0019219) (Figure 4B). Based on the GO enrichment analysis, 21 genes involved in the immune response were analyzed further for the enriched KEGG pathways. As shown in Figure 4C and Table S8, the top three highest hits of the immune-related signaling pathways were the NF-κB signaling pathway (10 DEGs), MAPK signaling pathway (9 DEGs), and transforming growth factor-β (TGF-β)-signaling pathway (9 DEGs) [19]. Additionally, the antigen processing and the presentation signaling pathways were also enriched in KEGG pathways. Further details of the expression values of immune-related DEGs involved in the antigenic presentation and signaling pathways are presented in Table 1.

**Figure 5:**
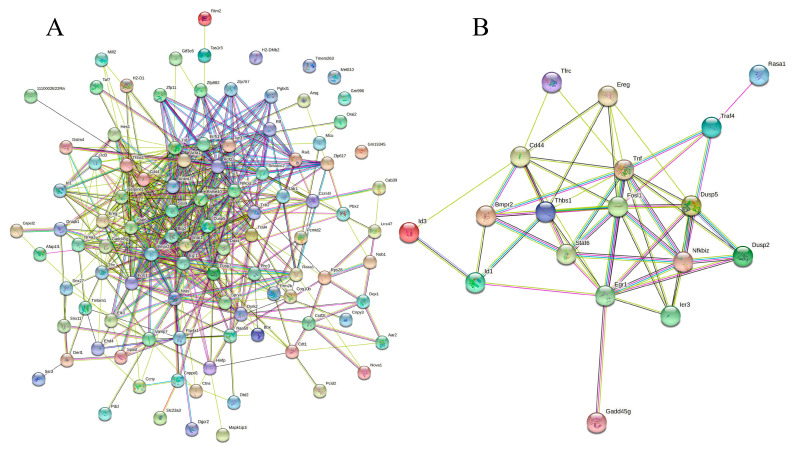
Protein−protein interaction network of all the DEGs (**A**) and immune-related genes. (**B**) PPI enrichment *p*−value: < 1.0 × 10^−16^. (**A**) In the PPI map, each small circles with 3D molecules filled represents different proteins in the network. The different colors of the attachment mean different interactions, including gene neighborhood, gene fusions, gene co-occurrence, and gene co-expression, supported by experimental determination or database mining. (**B**) A total of 19 nodes and 54 edges were included, with an average node degree of 5.68 and an average local clustering coefficient of 0.634 (*p* < 1.0 × 10−16). The PPI network shows that TNF (tumor necrosis factor) was the most prominent (primary) hub with 13 related proteins, and Egr1 and Fosl1 are the secondary hubs with 11 associated proteins each (details are provided in Table S9).

Next, the 139 DEGs were subjected to PPI analysis to investigate the mode of interaction in mice. The PPI network (*p* < 1.0 × 10^−16^) demonstrated that 100 proteins interacted with one or more proteins, as illustrated in Figure 5A. In total, 506 events of protein–protein interactions were established using STRING [20]. A new PPI network (*p* < 1.0 × 10^−16^) was established for the 20 DEGs involved in the immune response underpinning the complicated anti-inflammatory mechanism of rice biopeptides (Table 1), including antigen processing and presentation, the JAK-STAT signaling pathway, the MAPK signaling pathway, the TGF–β signaling pathway, the NF-κB signaling pathway, and the other cancer-related signaling pathways (Figure 5B). Details of the interaction analysis via STRING are provided in Table S9.

### 2.4. Validation of DEGs of the PEP1 Peptide−Stimulated DC2.4 Cells

**Figure 6:**
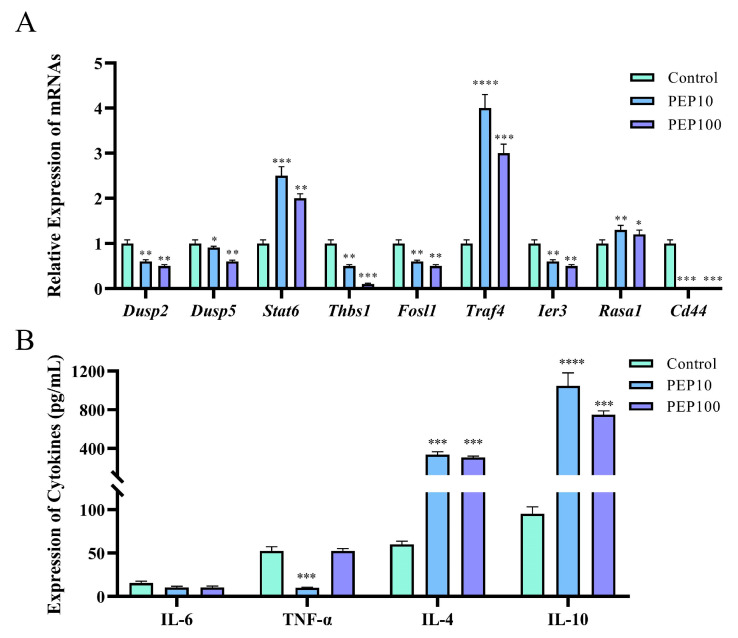
The validations of immune response induced by PEP1 peptide. (**A**) Gene expression of 9 DEGs via RT-qPCR. (**B**) The contents of pro-inflammatory and anti-inflammatory factors determined via ELISA. * p < 0.05 and ** *p* < 0.01 *** *p* < 0.001 and **** *p* < 0.0001 compared with the control group.

To investigate whether or not the PEP1 peptide stimulated the immune regulation of DC2.4 cells, nine DEGs involved in signaling pathways were analyzed via RT-qPCR, and cytokine production was assessed using an ELISA. As shown in Figure 6A, six genes were down-regulated via PEP1 stimulation, including *Dusp2* (dual specificity phosphatase 2), *Dusp5* (dual specificity phosphatase 5), *Thbs1* (thrombospondin 1), *Fosl1* (fos−like antigen 1), *Ier3* (immediate early response 3), and *Id3* (inhibitor of DNA binding 3). Three genes were upregulated (both *p <* 0.05), including *Stat6* (signal transducer and activator of transcription 6), *Traf4* (TNF receptor-associated factor 4), and *Rasa1* (RAS p21 protein activator 1). The RT-qPCR results confirmed the results of transcriptomic analysis, except for the expression of the *Ier3* gene, indicating the changes in the DEGs were obtained through transcriptomic analysis [18]. Interestingly, the comparison showed that a series of pro-inflammation related signal transduction genes were down-regulated, whereas those anti-inflammation- and tumor suppression-related genes were upregulated.

### 2.5. Anti-Inflammatory Activity of the PEP1 Peptide

The gene function analysis indicated that genes involved in pro-inflammatory and anti-inflammatory responses were differentially expressed in the PEP1 peptide-stimulated groups, compared to those in the blank control group. Therefore, the pro-inflammatory factors (IL-6 and TNF-α) and anti-inflammatory factors (IL-4 and IL-10) were determined using the ELISA method. As shown in Figure 6B, no significant changes in IL-6 were found in the 10 μg/mL and 100 μg/mL PEP1-treated groups, whereas TNF-α decreased significantly in both the Pep10 and Pep100 groups (both *p <* 0.01). In addition, it was found that in the Pep10 and Pep100 groups, the concentrations of IL-4 and IL-10 were significantly higher than those in the control group (*p* < 0.01). The observed changes in the PEP1-treated groups (decrease in the pro-inflammatory factor TNF-α and increases in the anti-inflammatory factors IL-4 and IL-10) implied that PEP1 peptide treatment contributes to the down-regulation of inflammatory responses and the upregulation of anti-inflammatory responses [16,21].

### 2.6. In Silico Prediction of PEP1 Binding Capability

**Figure 7:**
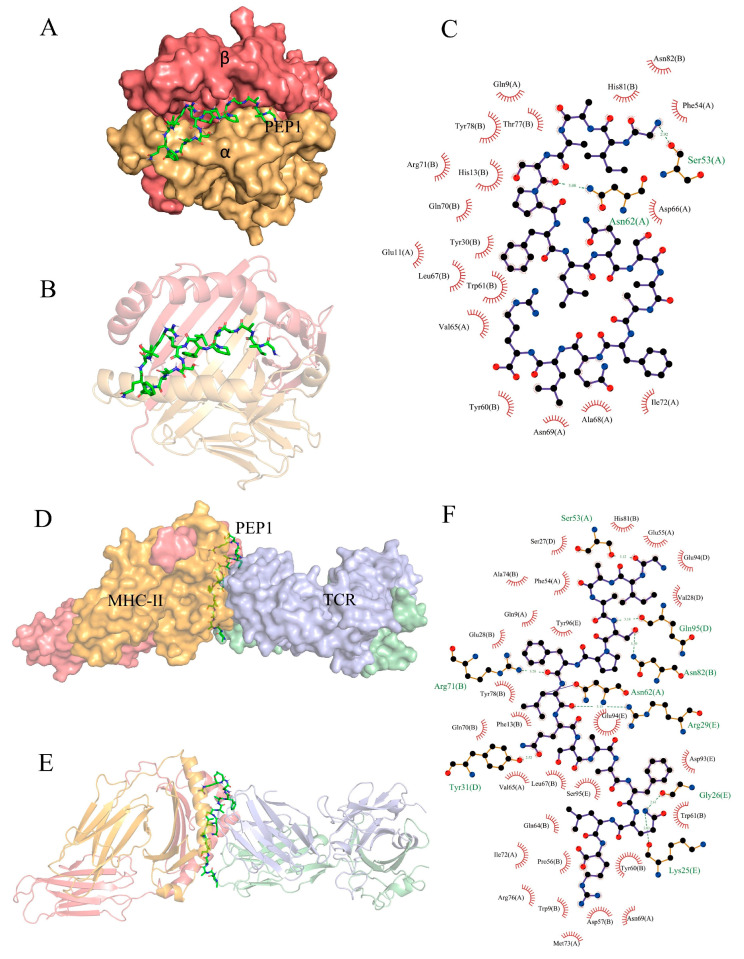
In silico docking of the PEP1 peptide with the targets. (**A**,**B**) The combined spatial simulation of the PEP1–MHC-II complex. (**C**) The contact details of the interface between the PEP1 and MHC-II molecule. (**D**,**E**) The combined spatial simulation of the PEP1–MHC-II–TCR triplet complex. (**F**) The contact details of the interface between the PEP1 and MHC-II–TCR complex.

Partial exogenous peptides can bind to the α- and β-chain grooves in the MHC-II molecule to form a complex and can also be recognized by TCRs [22]. A TCR consisting of α- and β-chains engages the peptide–MHC-II complex in the usual interleaved pattern, with the peptide in the middle of the two molecules, the β-chain of the TCR in contact with the MHC-II α-chain, and the α-chain in contact with the MHC-II β-chain [23]. AutoDock Vina can facilitate the execution of both simple and complex docking simulations by providing Python binding [24]. The results are shown in Figure 7. PEP1 peptide showed low combination energy to both the MHC-II molecule and MHC-II/TCR molecule, namely −10.4 kcal mol^−1^ and −6.2 kcal mol^−1^, respectively. The PEP1 peptide formed two hydrogen bonds with the amino acid residues Ser53 and Asn82 of MHC-II and electrostatic interactions with Arg72. Four hydrogen bonds were formed between the PEP1 peptide and the amino acid residues Gln95, Arg29, Tyr31, and Lys25 of the TCR molecule. Molecular docking confirmed that the PEP1 peptide can theoretically bind to MHC-II molecules, and then the PEP1–MHC-II complex can bind with the TCR molecule. Finally, a PEP1–MHC-II–TCR triplet complex was formed.

## 3. Discussion

In our previous study, it was proven that rice-derived peptide PEP1 shows strong immunoregulatory activity [15]. However, the underlying mechanisms of the PEP1 peptide’s involvement in immunoregulatory responses and immune-related signal pathways is still unclear. DCs are the most effective APCs that can be recognized by extracellular immunopeptides and bound by MHC-II molecules; this complex can present on the surface of the APCs and bind to the TCR on T-cells to activate the immune response [25,26]. Therefore, cell phenotype analysis of DC2.4 and the transcriptomic study were used to uncover the mechanism of the PEP1 peptide. The application of ISO-Seq can overcome the read length issues inherent to RNA-Seq methods. The long sequencing reads obtained from the PacBio sequencing platform can minimize the number of erroneous assemblies and has the advantages of long reading, high throughput, easy operation, and the property of being quantifiability [18]. A high-quality full-length transcriptome of DC2.4 cells was generated via PacBio ISO-Seq, which enabled a comprehensive insight into the immune response to food-derived peptides. Based on GO and KEGG analysis, many genes were involved in the activation of anti-inflammatory and antitumor responses, which were the most significant characteristics of the immune response in DC cells associated with PEP1 peptide incubation (Figure 4 and Table 1).

Previous research has confirmed that DCs are directly involved in the regulation of innate immunity and have an important role in bridging innate and adaptive immunity. Antigen capture can induce the maturation of APCs and the expression of surface MHC molecules, and co-stimulatory molecules (including CD80 and CD86). It can also enhance the antigen presentation ability of APC [27,28]. Our results confirmed that the PEP1 peptide can activate the maturation of mouse DC2.4 cells, as well as stimulate the expression of CD86 and MHC-II markers (Figure 1). The DCs can present the antigenic peptides and stimulate CD4^+^ T-cells, but MHC-II molecules must be loaded with extracellular peptides first; this step is governed by the MHC-II-like H2-M molecules (H2-M/DM), and H2-M/DM functions as a peptide editor that serves to positively select peptides that can stably bind to MHC-II molecules [27]. From the RNA-seq results, the expression of the *H2-DMb2-3* gene was upregulated (Table 1), which has been shown to contribute to the antigen loading of the MHC-II molecule subunit [29]. In addition, the molecular docking results of the PEP1–MHC-II–TCR triplet complex show that PEP1 forms hydrogen bonds and electrostatic interactions with MHC-II and TCR, binding stably with low binding energy. A study showed that the conserved amino acid residue site N82 of the β-chain of MHC-II contributes to the stability of the peptide/MHC-II and could be involved in controlling peptide accommodation [30]. In this study, a hydrogen bond was found between the Ser5 residue of PEP1 and Asn82 of MHC-II in the simulated PEP1–MHC-II–TCR triplet complex (Figure 7). These results suggest that the PEP1 peptide can stimulate DC2.4 cell maturation and endows it with a high affinity to the target protein (MHC-II and TCR), which may be involved in antigen presentation. However, these results need to be further confirmed.

**Figure 8:**
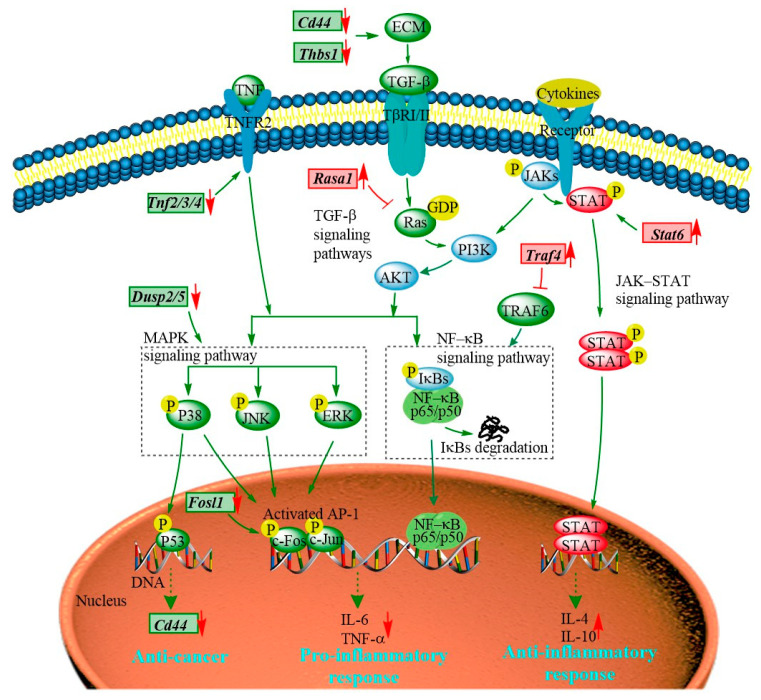
The hypothetical signaling network stimulated by the PEP1 peptide in mouse DC2.4 cells.

The transcriptomic results show that the majority of the DEGs regulated by the PEP1 peptide are enriched in JAK-STAT, MAPK, TGF–β, and NF-κB signaling pathways (Figure 8 and Table S8). The JAK-STAT signaling pathway is essential for initiating intrinsic immunity and coordinating adaptive immunity; an important member of this pathway is the STAT6 protein, which can activate the gene expression of anti-inflammatory factors and is involved in IL-4/IL-13-mediated signaling [31]. Subsequently, it can regulate the downstream signaling transduction of the PI3K/AKT and MAPK pathways [32]. The *Stat6* gene was found in PEP1 peptide-stimulated DEGs and analyzed via RT-qRCR (Table 1 and Figure 6). The MAPK signaling pathway is also closely related to the inflammatory response, including three key pathways via ERK (extracellular regulated kinase), JNK (Jun N-terminal kinase), and p38 [19]. Dual-specificity phosphatase 2 is a known regulator of ERK and p38 MAPKs, and it is able to bind with ERK3/4 and down-regulate via PEP1 peptide stimulation (Table 1 and Figure 6). The absence of DUSP2 can protect mice against obesity-associated inflammation [33]. Therefore, DUSP2 can modulate inflammatory responses by negatively regulating ERK and p38 activity in vitro [34]. Another dual-specificity phosphatase (DUSP5) was also down-regulated by the PEP1 peptide. Down-regulating DUSP5 can rescue the level of ERK phosphorylation and negatively regulate ERK signaling [35]. Thus, we hypothesized that the down-regulation of DUSP2/5 could inhibit the NF-κB signaling pathway via ERK and suppress the expression of pro-inflammatory cytokines (Table 1 and Figure 6). In addition, proteins of the tumor necrosis factor receptor-associated factor (TRAF) family play key roles in a variety of biological processes, including immunity, inflammation, and apoptosis [36]. Interestingly, in the present study, PEP1 treatment up-regulated the *Traf4* gene (Table 1 and Figure 6), thereby inhibiting the action of TRAF6, whereas the down-regulation of the *Fosl1* gene expression resulted in the down-regulation of the AP-1 protein and ultimately suppressed the production of pro-inflammatory factors. The PEP1 peptide regulates the expression of these genes by inhibiting the activation of the NF-κB signaling pathway, thereby recruiting pro-inflammatory *Tnf* gene expression (Table 1 and Figure 8). Thus, changes in the expression of genes involved in the JAK-STAT signaling pathway and MAPK signaling pathway due to the stimulation of the PEP1 peptide inhibited the NF-κB signaling pathway, resulting in a decrease in pro-inflammatory factors (TNF-α) and an increase in anti-inflammatory factors IL-4 and IL-10 (Table 1 and Figure 6). This suggests that the PEP1 peptide has potential anti-inflammatory capacity.

In addition to the DEGs associated with anti-inflammation activity via the MAPK/NF-κB signaling pathway, several DEGs associated with antitumor activity were also involved in the MAPK signaling pathway (Table 1 and Figure 8). RASA1 (Ras GTPase-activating protein 1) is a regulator of Ras GDP and GTP that promotes the pathological processes of vascular disease and tumor formation [37]. RASA1 acts as a direct regulator of the Ras−MAPK pathway protein affecting its downstream genes. The *Rasa1* gene was found to be up-regulated in response to PEP1 peptide treatment, suggesting that PEP1 contributes to anti-tumorigenicity (Table 1 and Figure 6). The expression of RASA1 can be down-regulated by miRNA, which promotes cell proliferation and inhibits liver cancer cell apoptosis. Thus, RASA1 is an attractive target for tumor treatment [37]. The TGF−β signaling pathway has a key role in cell growth, migration, differentiation, epithelial–mesenchymal transition, and extracellular matrix remodeling [38]. The expression of many genes involved in the TGF−β signaling pathway is also regulated by the PEP1 peptide. The zinc finger transcription factor EGR1 has been shown to activate the expression of TGF−β1 and p53, thus contributing to the prevention of tumor formation. In the present study, the expression of the EGR1 (early growth response protein 1) gene was found to be up-regulated by the PEP1 peptide [39]. The *Thbs1* gene, encoding an extracellular matrix (ECM) protein thrombospondin−1 (THSB1) was found to be up-regulated by the PEP1 peptide (Table 1 and Figure 6) [40]. Fibrosis caused by the excessive deposition of proteoglycans such as THSB1 in the ECM provides the conditions for tumor cell migration [41]. In addition, the PEP1 peptide has a significant down-regulatory effect on *Cd44* gene expression [38]. The adhesion molecule CD44 is a cell surface type 1 hyaluronan transmembrane glycoprotein receptor, and the inhibition of CD44 signaling has been found to be beneficial for the treatment of tumor patients [5]. Here, the CD44 molecule was found co-expressed with other proteins, including STAT6, THSB1, and TNF molecules in mice. Moreover, both *Cd44* and *Thsb1* genes were involved in the ECM−receptor interaction KEGG pathway (Table S8). In the present study, the expression of *Thbs1*, *Tnfs*, and *Cd44* was down-regulated (Table 1 and Figure 6), indicating the potential tumor suppression function of the PEP1 peptide. These key genes were validated via RT-qPCR and the results were basically accordant with the results of transcriptomic analysis (Figure 6). Thus, the antitumor capacity of peptide PEP1 is preliminarily proposed in the present study. Whereas, further investigations are still needed to refine the mechanism of PEP1 anticancer activity.

## 4. Materials and Methods

### 4.1. Chemicals and Reagents

Fetal bovine serum (FBS) and trypsin were purchased from Hangzhou Sijiqing Biology Engineering Materials Co. (Hangzhou, China). Anhydrous p−amino benzenesulfonic acid, dimethyl sulfoxide (DMSO), 3−(4,5−dimethylthiazol−2−yl)−2,5−diphenyltetrazolium bromide (MTT), *N*−(1−naphthyl) ethylenediamine dihydrochloride, and PCR primers were purchased from Sangon Biotech (Shanghai, China). RPMI 1640 and Gibco penicillin−streptomycin (10,000 units/mL streptomycin and 10,000 units/mL penicillin) were purchased from Thermo Fisher Scientific (Shanghai, China). TRIzol reagent was purchased from Thermo Fisher Scientific (Shanghai, China). M−MLV reverse transcriptase was procured from Takara Bio Inc. (Beijing, China). Mouse IL-4/IL-6/IL-10/TNF-α ELISA kits were purchased from DAKEWE (Shanghai, China). The PEP1 peptide (GIAASPFLQSAAFQLR) with a purity of >95% was synthesized by Jill Biochemical Company (Shanghai, China).

### 4.2. Cell Culture

The mice dendritic cell line (DC2.4) was obtained from Wuhan Mingzhou Biotechnology Co. The DC2.4 cells were cultured in RPMI 1640 (Gibco, Shanghai, China) supplemented with 10% fetal bovine serum (FBS, Gibco, Shanghai, China) and penicillin–streptomycin (10,000 U/mL penicillin and 10,000 U/mL streptomycin) (PS, Gibco, Shanghai, China). All cells were cultured at 37 °C in a humidified atmosphere with 5% CO_2_ and subcultured every other day. DC2.4 cells were incubated with the PEP1 peptide at 1, 10, and 100 μg/mL for 24 h and the effect on the cells was investigated via the MTT assay, flow cytometry, and confocal imaging. A blank was used as the control.

### 4.3. In Vitro DC2.4 Cell Viability Assay

The viability of DC2.4 cells treated with the PEP1 peptide was determined via the MTT assay in accordance with the manufacturer’s suggestions. Briefly, DC2.4 cells were seeded in two 96−well plates at a density of 1.5 × 10^4^ cells per well, and cultured for 24 h in 1, 10, or 100 μg/mL of PEP1 peptide solutions, named as groups Pep1, Pep10, and Pep100 (*n* = 6 in each group). Sterile water was used as the control treatment. After treatment, the medium was removed and replaced with a RPMI 1640 solution containing 20 µL of MTT (5 mg/mL). Then, the DC2.4 cells were cultured for 4 h in a cell incubator. Thereafter, the culture medium was removed, 150 μL of DMSO was added to each well, and the entire plate was shaken at 25 °C until the solid material was completely dissolved. Cell viability was determined by measuring the absorbance at 490 nm with a microplate reader (µQuant, BIO-Tek, VT, USA), and calculated based on the ratio of the optical density (OD) value of the peptide-treated cells to the OD value of the control cells. All experiments were repeated three times.

### 4.4. Detection of Surface Characteristic Molecules on DC2.4 Cells

DC2.4 cells were pre-incubated with different concentrations of peptide PEP1 to stimulate the secretion of factors MHC−II and CD86. DC2.4 cells subcultured in peptide PEP1 solutions at a concentration of 0, 1, 10, or 100 μg/mL for 24 h were collected. The medium was discarded and the cells were detached using a cold Trypsin−EDTA solution; the cells were collected and centrifuged at 1000 × *g* for 5 min. The cells were washed three times and resuspended in PBS buffer, then stained with allophycocyanin (APC)-conjugated anti-mouse CD86 (eBioscience, CA, USA; clone: GL1), PE-conjugated anti-mouse MHC-II (I−A/I−E) (eBioscience, CA, USA; clone: M5/114.15.2), or isotype controls for 30 min at 4 °C in the dark. After washing the cells three times with PBS, a flow cytometer (BD Accuri C6 Plus) was used to quantify the labeled cells, and the Accuri C6 Plus and CFlow software was used for data analysis. The isotype monoclonal antibodies for the two types of monoclonal antibodies were used as background controls in addition to unstained cells.

### 4.5. DC2.4 Cell Morphology

DC2.4 cells at a density of 1 × 10^5^ cells/cm^2^ were cultured in the absence and presence of the PEP1 peptide for 24 h. An FV3000 Olympus laser confocal microscope was used to visualize the stimulatory effect of the PEP1 peptide on DC2.4 cells using only bright-field imaging. The morphology of immature and mature DCs had different characteristics.

### 4.6. ISO-Seq Library RNA Extraction, Detection, Sequencing, and Analysis

Based on the results of the above experiments, the control, Pep10, and Pep100 groups were investigated via transcriptomic analysis. DC2.4 cells were cultured in 6-well plates at a density of 3 × 10^5^ cells per well in 2 mL of RPMI 1640 containing 10% FBS. After 12 h, DC2.4 cells were incubated with different concentrations of the PEP1 peptide (0, 10, and 100 μg/mL) for 24 h. The cells were then collected, and total RNA was extracted from samples using a Qiagen kit (USA). Total RNA samples with 2.0 < OD_260/280_ < 2.2 and 1.8 < OD_260/230_ < 2.1 as analyzed using the NanoDrop spectrophometer were used for constructing the cDNA libraries in PacBio and Illumina sequencing. RIN (RIN ≥ 8) and 28S/18S values (28S/18S ≥ 1.5) were detected using Agilent 2100 Bioanalyzer (Agilent, CA, USA). Sequencing was performed on the Pacific Bioscience Sequel platform (Pacific Biosciences, CA, USA), and three SMRT cells were run. The full-length sequence data of mouse DC2.4 cells (control, Pep10, and Pep100 groups) have been submitted to the NCBI database under accession numbers from SAMN28795843 to SAMN28795845 (Reviewer link: https://dataview.ncbi.nlm.nih.gov/object/PRJNA844180?reviewer=1v0r89nek90a5utb3gha1mbll2, accessed on 1 June 2022). After sequencing, high-quality sequencing data were obtained through filtering. Subsequently, the original data were processed using SMRTLink 8.0 analysis software. The transcriptome was assembled and corrected, and then annotated by comparing the ISO-Seq transcripts with the reference genome annotations. More details are provided in the Supplementary Methods.

### 4.7. RNA-Seq Library RNA Preparation, Sequencing, and Analysis

The Illumina library was prepared using NEBNext^®^Ultra^TM^ RNA Library Prep Kit for Illumina^®^ (NEB, USA) in accordance with the manufacturer’s instructions, and Illumina NovaSeq 6000 (Illumina Inc., CA, USA) was used for library sequencing and generating paired−end reads. The sequence data of nine samples were archived at NCBI database under the accession numbers from SAMN28795834 to SAMN28795842 (Reviewer link: https://dataview.ncbi.nlm.nih.gov/object/PRJNA844180?reviewer=1v0r89nek90a5utb3gha1mbll2, accessed on 1 June 2022). More details about the bioinformatic analysis combining ISO-Seq and RNA-Seq are provided in the Supplementary Methods (Figure S1). The data from the Illumina platform were used for the quantitative and differential expression analysis of all the transcripts, using StringTie v2.2.0 software. The counts of the reads were transformed into FPKM (fragments per kilobase of transcript per million fragments mapped) to estimate the gene expression levels. The correlation coefficients of gene expression between samples (different groups and the biological duplicates) were analyzed. The FPKM box of different groups was used to measure the differences among samples from the perspective of the overall dispersion of expression quantity. Based on the amount of gene expression, the principal component analysis (PCA) method was used to determine gene expression differences between the samples, which allowed the determination of the sample clustering relations.

### 4.8. Annotation and Function Analysis

ANGEL 2.4 was applied to predict the coding sequences (CDSs) and protein open read frames (ORFs). For comprehensive functional annotation, the non-redundant transcripts were annotated based on the following databases: NR (NCBI non-redundant protein sequences), Swiss-Prot (https://www.expasy.org/resources/uniprotkb−swiss−prot, accessed on 1 June 2022), GO (Gene Ontology, http://www.geneontology.org, accessed on 1 June 2022), KEGG (Kyoto Encyclopedia of Genes and Genomes, https://www.genome.jp/kegg, accessed on 1 June 2022), and KOG/COG (Cluster of Orthologous Groups of proteins, http://www.ncbi.nlm.nih.gov/COG/, accessed on 1 June 2022). The ClusterProfiler software 3.10.0 was used for function enrichment analysis (*p*-value < 0.05), and the functional information was assigned to the best-matched sequence.

### 4.9. Analysis of the Differentially Expressed Genes (DEGs)

Differential expression for three duplicated samples of the control, Pep1, and Pep100 groups was analyzed using DESeq2 software 1.20. Genes with a *p*-value of ≤0.01 were corrected by the false discovery rate (FDR) of 0.05; therefore, genes with *FDR* ≤ 0.05 were considered to be statistically significant and then filtered for subsequent analysis.

STRING 11.5 (https://cn.string−db.org, accessed on 1 June 2022), an online site for building visual networks of protein–protein interactions (PPI), was used to find the relationship between the significant DEGs. A network map was constructed for the DEGs, and the mode of interaction and functional analysis were also provided [20].

### 4.10. qPCR Analysis of Selected Genes

Based on the PPI analysis, nine genes (*Dusp2*, *Dusp5*, *Thbs1*, *Traf4*, *Fosl1*, *Ier3*, *Stat6*, *Rasa1*, and *Cd44*) were selected and most of them are closely related to signaling pathways. RT-qPCR was conducted according to a previously reported method [18], while DC2.4 cells (3.5 × 10^5^ cells per well) were seeded onto a 96-well plate, incubated for 12 h, and then pretreated with the PEP1 peptide at various final concentrations (0, 10, and 100 µg/mL) for 24 h. Next, TRIzol reagent was added to the DC2.4 cells to extract total RNA. Total RNA (2 µg) was reverse-transcribed to first-strand cDNA using M−MLV reverse transcriptase. The cDNA was amplified using murine-specific primers designed with Primer Premier 5 (Table S1) and more details of the RT-qPCR analysis are shown in the Supplementary Methods.

### 4.11. Determination of Pro-Inflammatory and Anti-Inflammatory Factor Concentration via ELISA

DC2.4 cells were seeded onto 24-well culture plates at a density of 1.5 × 10^5^ cells per well and incubated for 24 h. The cells were subsequently treated with three concentrations of the PEP1 peptide (0, 10, and 100 µg/mL) for 24 h. The supernatants were collected, and the concentrations of pro-inflammatory (IL-6 and TNF-α) and anti-inflammatory (IL-4 and IL-10) factors in the medium were analyzed using mouse ELISA kits (DAKEWE, China) in accordance with the manufacturer’s instructions. A microplate reader at 450 nm was used to measure the absorbance, and the concentration of each cytokine was calculated using the standard curve provided by the manufacturer.

### 4.12. In Silico Docking of the PEP1 Peptide and Targets

The PEP1 peptide was docked to the crystal structure of the MHC-II molecule (PDB ID: 6BIY) and MHC-II−TCR complex (PDB ID: 6R0E) using the AutoDock Vina software (http://vina.scripps.edu) [42,43]. The structures of the PEP1 peptide, MHC-II molecule, and MHC-II−TCR complex were converted into the PDBQT format using AutoDock Vina software. A grid box covered the α1 chain and β1 chain of the MHC-II molecule. The planar and stereoscopic interactions between the receptor ligand and peptide were analyzed using PyMOL.

### 4.13. Statistical Analysis

Three independent experiments were performed for each analysis, and the results were presented as the mean ± standard deviation of the three experiments. Statistically significant differences in the results were analyzed via a one-way analysis of variance (ANOVA). The statistical software SPSS 22.0 was used, and *p*-values of less than 0.05 were considered to indicate statistical significance.

## 5. Conclusions

In summary, the molecular mechanism of rice-derived immunopeptide PEP1 was deeply investigated in this study. PEP1 can stimulate DC2.4 cell maturation and up-regulate the expression of the characteristic cell surface molecules MHC-II and CD86, thereby enhancing anti-inflammatory and anti-cancer abilities. Genes involved in the pro-inflammatory responses were inhibited, leadings to increased anti-inflammatory factors and reduced pro-inflammatory factors. The findings revealed that the PEP1 peptide could potentially be used as a natural alternative anti-inflammatory therapy. Moreover, its potential anti-tumor activity was also revealed via transcriptomic analysis. The actual effect of the PEP1 peptide needs to be further confirmed in animal experiments or clinical trials. In conclusion, our study lays a theoretical foundation for the potential pharmaceutical application of rice-derived immunopeptides.

## Figures and Tables

**Table 1:** The expression values of DEGs involved in the antigenic presentation and signaling pathways.

Gene ID	Gene Description	Control	Pep10	Pep100
*Bmpr2*	Bmpr2, bone morphogenetic protein receptor type II	2.72 ± 0.06	2.96 ± 0.05 **	4.41 ± 0.69 *
*Dusp2*	Dusp2, dual specificity phosphatase 2	8.61 ± 0.10	5.66 ± 0.61 **	4.3 ± 0.39 **
*Dusp5*	Dusp5, dual specificity phosphatase 5	24.61 ± 1.18	16.23 ± 1.74 **	9.27 ± 1.27 **
*Ereg*	Proepiregulin, ligand of the EGF receptor/EGFR and ERBB4	3.44 ± 0.21	2.87 ± 0.12 *	1.56 ± 0.28 **
*Fosl1*	Fosl1, fos−like antigen 1	35.92 ± 2.29	31.91 ± 0.91 *	25.63 ± 0.61 **
*Gadd45g*	Growth arrest and DNA−damage−inducible 45 gamma	191.19 ± 17.40	119.09 ± 23.73 *	91.49 ± 22.53 **
*Gm13872*	Cd44, CD44 antigen	0.07 ± 0.02	0.01 ± 0.01 *	0.00 ± 0.00 **
*H2−DMb2−3*	H2−DMb2, histocompatibility 2, class II, locus Mb2	0.90 ± 0.02	2.61 ± 0.59 **	3.34 ± 0.55 **
*Id1*	Id1, inhibitor of DNA binding 1	139.12 ± 7.69	69.36 ± 12.66 **	20.20 ± 7.56 **
*Id3*	Id3, inhibitor of DNA binding 3	70.16 ± 9.58	29.85 ± 3.7 **	13.34 ± 3.35 **
*Ier3*	Ier3, immediate early response 3	201.95 ± 19.83	128.79 ± 12.79 **	96.16 ± 9.66 **
*Nfkbiz*	Nfkbiz, nuclear factor of kappa light polypeptide gene enhancer in B cells N/A inhibitor, zeta	20.64 ± 1.85	15.54 ± 2.26 *	11.00 ± 1.15 **
*PB.11742*	Traf4, TNF receptor−associated factor 4	0.12 ± 0.02	0.61 ± 0.13 **	0.45 ± 0.02 **
*PB.16988*	Egr1, early growth response protein 1	1.18 ± 0.19	1.12 ± 0.07	2.76 ± 0.66 *
*PB.13686*	Rasa1, RAS p21 protein activator 1	0.59 ± 0.03	0.82 ± 0.06 **	0.66 ± 0.05
*Stat6*	Stat6, signal transducer and activator of transcription 6	23.37 ± 1.69	32.79 ± 1.11 **	32.54 ± 1.97 **
*Thbs1*	Thbs1, thrombospondin 1	3.24 ± 0.24	1.8 ± 0.33 **	1.09 ± 0.40 **
*Tnf-2*	Tnf2, tumor necrosis factor 2	1.05 ± 0.02	0.50 ± 0.06 **	0.45 ± 0.03 **
*Tnf-3*	Tnf3, tumor necrosis factor 3	1.35 ± 0.15	0.95 ± 0.02 **	0.55 ± 0.09 **
*Tnf-4*	Tnf4, tumor necrosis factor 4	1.26 ± 0.05	0.81 ± 0.21 *	0.50 ± 0.04 **

* *p* < 0.05; **p < 0.01.

## Data Availability

The article contains data supporting the findings.

## Supplementary Materials

The following supporting information can be downloaded at https://www.mdpi.com/article/10.3390/molecules28135224/s1. Figure S1: Flow diagram for ISO-Seq and RNA-Seq; Figure S2: ISO-Seq sequencing, assembly, and annotation of mouse DC2.4 cells; Figure S3: GO enrichment of the 35 DEGs regulated by 10 µg/mL and 100 µg/mL of the PEP1 peptide; Table S1: Primers for reverse-transcription PCR; Table S2: Summary of sample post-filter polymerase reads; Table S3: Statistics of CDS in novel transcripts and novel genes; Table S4: Summary of annotation results; Table S5: Results of comparison with reference genome; Table S6: The expression levels of all genes; Table S7: 139 DEGs including FPKM, GO and KEGG (in a separate XLSX sheet); Table S8: KEGG enrichment of genes involved in immune response; Table S9: I Interaction analysis via STRING.
