# Supplementary material for: Transcriptome Analysis Reveals the Immunoregulatory Activity of Rice Seed-Derived Peptide PEP1 on Dendritic Cells
Source: Molecules. 2023 Jul 5;28(13):5224. doi: 10.3390/molecules28135224 (PMC10343632; doi:10.3390/molecules28135224)
Supplement: Supplementary file 1 [file molecules-28-05224-s001.zip › Supplementary materials.docx]

Supplementary Material

Transcriptome Analysis Reveals the immunoregulatory activity of rice seed-derived peptide PEP1 on dendritic cell

Tingmin Qu^1,†^, Shuwen He^1,†^, Ying Wu^1^, Yingying Wang^1^, Ce Ni^1^, Shiyu Wen^1^, Bo Cui^2^, Yunhui Cheng^1,2^, Li Wen^1,^*

^1^ Hunan Provincial Key Laboratory of Cytochemistry, School of Food Science and Bioengineering, Changsha University of Science & Technology, Changsha, 410114, China

^2^ School of Food Science and Engineering, Qilu University of Technology, Jinan, 250353, China

† Tingmin Qu and Shuwen He contributed equally to this work.

***** Correspondence: wl@csust.edu.cn

# Supplementary Methods

## SM1. ISO-seq Library RNA Extraction, Detection, Sequencing, and Analysis.

The DC2.4 cells were then collected, and total RNA was extracted from samples using Qiagen kit (USA). Total RNA samples with 2.0<OD 260/280 <2.2 and 1.8<OD 260/230 <2.1 detected by Nanodrop were used for constructing the cDNA libraries in PacBio and Illumina sequencing. RIN（RIN≥8）and 28S/18S values (28S/18S≥1.5) were detected by using Agilent 2100 Bioanalyzer.

To construct the library for PacBio sequencing, an equal quantity of RNA from three duplicated sample was pooled, and 3 pg total RNA was synthesized to cDNA and subsequently amplified to generate double-stranded cDNA using the Clontech SMARTer PCR cDNA Synthesis Kit (Takara, China). Size selection of the PCR product was performed using KAPA HiFi PCR Kits. Size Selection System (Sage science, USA), and the fragments with the length of 0.5-6 kb were retained. Each SMRTbell library was constructed using 1 pg size-selected cDNA with the Pacific Biosciences SMRTbell template prep kit 1.0 (USA). The binding of SMRT bell templates to polymerases was con­ducted using the Sequel Binding Kit (USA), and then primer annealing was performed. Sequencing was carried out on the Pacific Bioscience Sequel platform (USA), and three SMRT cells were run. The full-length sequence data of mice DC2.4 cells (Control, Pep10 and Pep100 groups) have been submitted to the NCBI database under the accession number from SAMN28795843 to SAMN28795845 (Reviewer link: https://dataview.ncbi.nlm.nih.gov/object/PRJNA844180?reviewer=1v0r89nek90a5utb3gha1mbll2).After sequencing, high-quality sequencing data were obtained through filtering. Subsequently, the original data was processed using SMRTLink 8.0 analysis software. The transcriptome was assembled using data from PacBio, and the polymerase reads were processed into error corrected reads of insert (ROIs) with min-Full Pass >1 and min-Predicted Accuracy >80%. All the ROIs were further classified into full-length (FL) and non-full-length (NFL) transcript sequences depend on whether the 5’ primer, 3’ primer and poly A tail were simultaneously observed. IsoSeq cluster software v3 (<https://github.com/PacificBiosciences/IsoSeq3>) was carried out for refining, correction and clustering the above data. Three-step strategies of error correction were employed to improve the accuracy of full-length transcripts produced by the ISO-Seq. Firstly, the circle sequencing with >1 passes provided the opportunity for circular consensus sequencing (CCSs) of self-correction. Secondly, full-length non-chimeric (FLNC) reads were subjected to non-redundant and cluster treatment by ICE Quiver algorithm and Arrow polishing with NFL sequence, herein high-quality and polished full-length consensus sequence were produced. Finally, the sequencing data was corrected by using IsoSeq3 Polish. The corrected isoforms were compared with reference genomes using GMAP software, and the fusion genes and LnRNAs were corrected. Finally, SQANTI2 software (https://github.com/Magdoll/SQANTI2) were used to obtain the clean isoforms without redundance, by comparing the ISO-Seq transcripts with the reference genome annotations.

## SM2. RNA-seq Library RNA Preparation, Sequencing, and Analysis.

The Illumina library was prepared using NEBNext®Ultra^TM^ RNA Library Prep Kit for Illumina® (NEB, USA) following the manufacturer's instructions. 1.5 ug RNAs of each sample was used for RNA-Seq library construction. Agencourt RNAClean XP beads (Beckman Coulter) was used to purify PCR products and select cDNA fragments of preferentially 150 bp in length. Illumina NovaSeq 6000 (Illumina Inc., CA, USA) was used for library sequencing and generating paired-end reads. The sequences data of nine samples were archived at NCBI database under the accession numbers from SAMN28795834 to SAMN28795842 (Reviewer link: <https://dataview.ncbi.nlm.nih.gov/object/PRJNA844180?reviewer=1v0r89nek90a5utb3gha1mbll2>). The polished consensus sequences by IsoSeq3 of ISO-seq were further subjected to correct and remove redundancy with Illunima short reads by using LoRDEC 0.9 tool. Full-length transcripts yielded by ISO-seq were used as reference se­quences, and the expression levels of all transcripts were further calculated based on the data generated by the Illumina sequencing. HISAT2 software was used to align the RNA-Seq sequences to sequences generated by ISO*-*seq, and the gene expression level was estimated using RSEM 1.2.15. The preprocessed reads were aligned to the reference sequences and the count of reads was obtained from the mapping results. The data of Illumina platform was used for quantitative and differential expression analysis of all the transcripts, by using StringtTie software. The counts of the reads were transformed into FPKM (fragments per kilobase of transcript per million fragments mapped) to estimate the gene expression levels. The correlation coefficients of gene expression between samples (different groups and the biological duplicates) were analyzed. The FPKM box of different groups were used to measure the differences among samples from the perspective of the overall dispersion of expression quantity. Based on the sample amount of gene expression, principal component analysis (PCA) method was used to determine gene expression differences between the samples, which can determine the sample clustering relations.

## SM3. qPCR Analysis of Selected Genes.

TRIzol reagent was added to the cells to extract total RNA. Two micrograms of total RNA was reverse-transcribed to first-strand cDNA by using M-MLV reverse transcriptase, and the cDNA was amplified using murine-specific primers designed with Primer Premier 5 (Table S1). RT-qPCR was performed according to a previously reported method [^1^](#_ENREF_1) by using an iCycler iQ system (Bio-Rad, CA, USA). Each reaction was conducted in a 20 μL PCR reaction solution (SsoAdvanced™ Universal SYBR^®^ Green Supermix, Bio-Rad, USA) with approximately 1.0 ng of the first-strand cDNA obtained from total RNA and 100 nM of each primer. Three technical repeats were used for each of the three biological duplicates. The PCR program was as follows: denaturation at 95°C for 45 s, followed by 40 cycles of 95°C for 20 s, 60°C for 20 s, and 72°C for 25 s. The ^ΔΔ^Ct method was used for RT-qPCR analysis, with the reference gene *β-actin* as an endogenous control.

References

Wen, L.; Li, W.; Parris, S.; West, M.; Lawson, J.; Smathers, M, et al. (2020). Transcriptomic profiles of non-embryogenic and embryogenic callus cells in a highly regenerative upland cotton line (Gossypium hirsutum L.). BMC Dev Biol, 20(1), 25.doi: 10.1186/s12861-020-00230-4

# Supplementary Figures and Tables

## Supplementary Figures

**
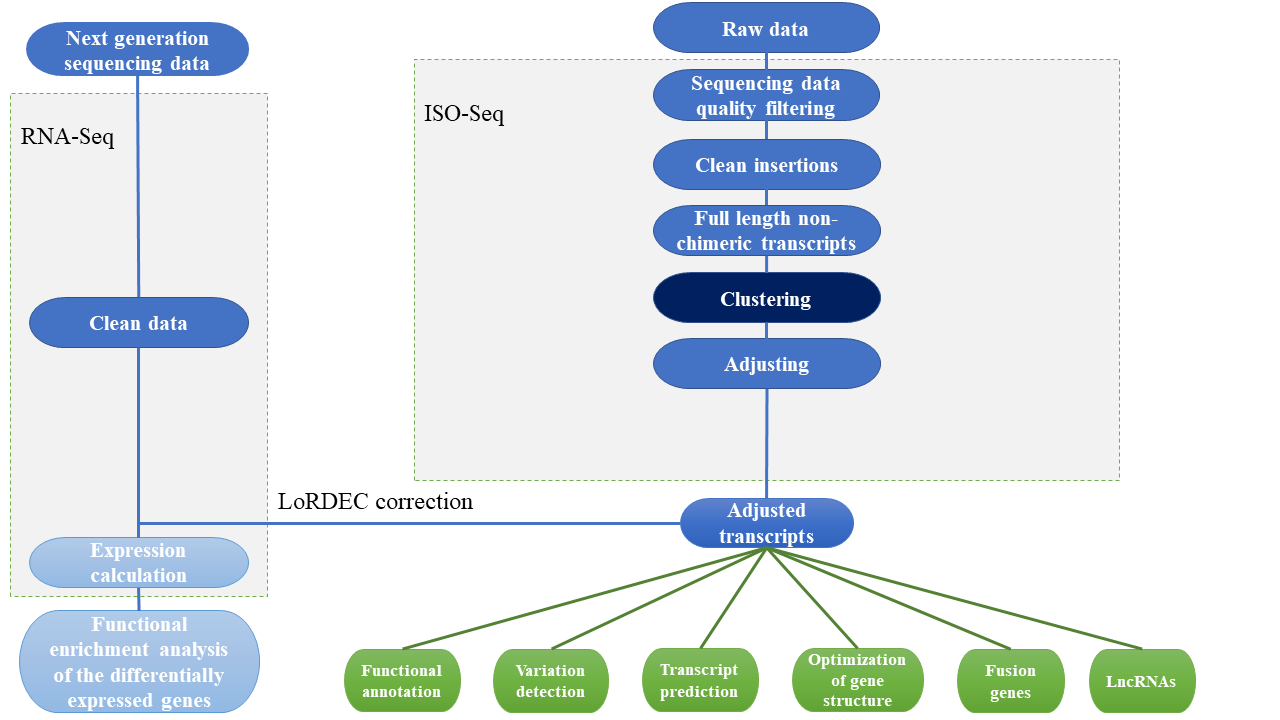
**

**Figure S1. Flow diagram for Iso-seq and RNA-seq**

**
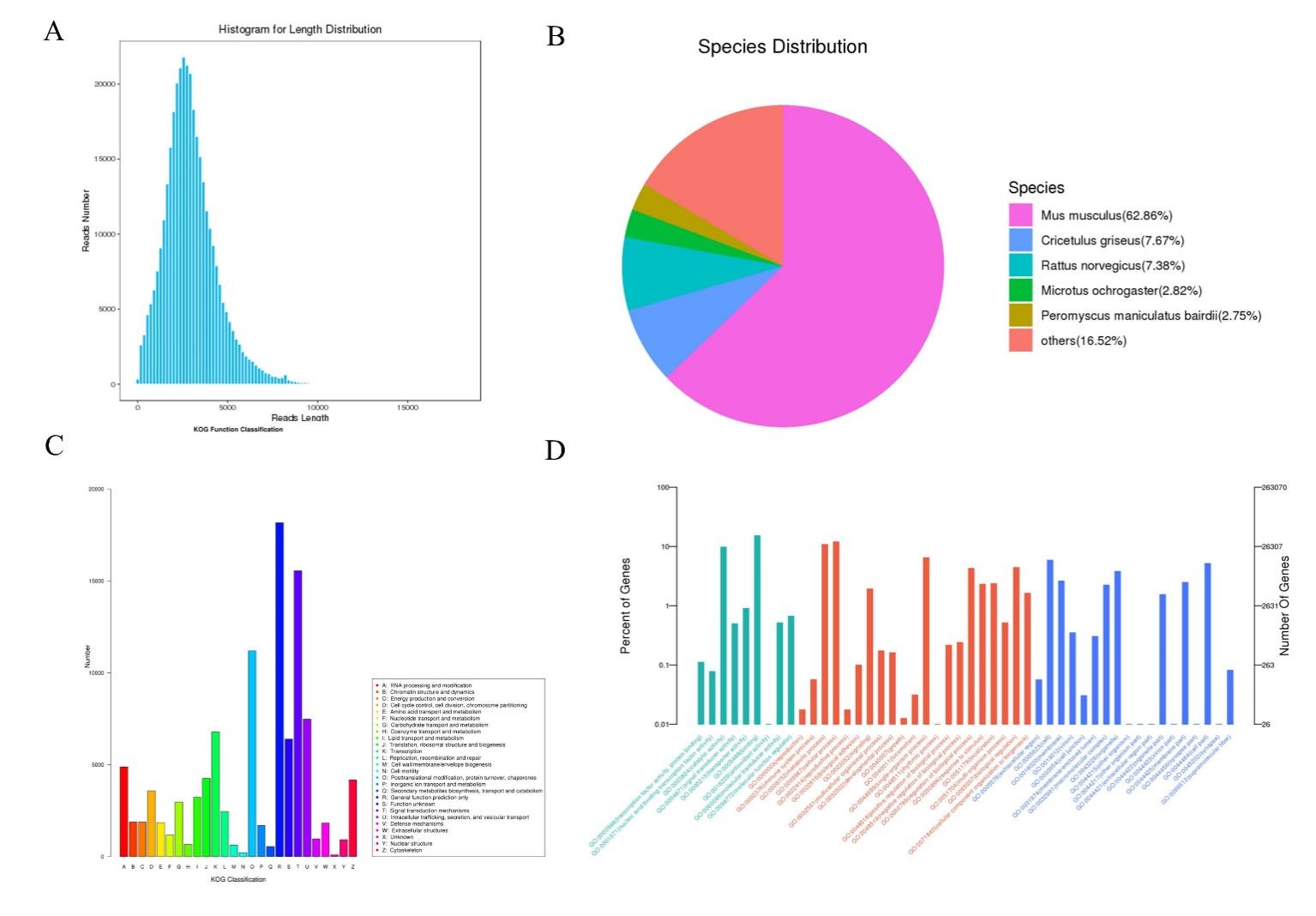
**

**Figure S2. ISO-Seq sequencing, assembly, and annotation of mice DC2.4 cells**

(A) Length distribution of the corrected sequences. (B) The distribution of homologous species annotated in the NCBI non-redundant protein (NR) database. (C) Cluster of Orthologous Groups of proteins (KOG) classification of the assembled full-length transcripts. (D) Gene Ontology (GO) classification of the assembled full-length transcripts.

**
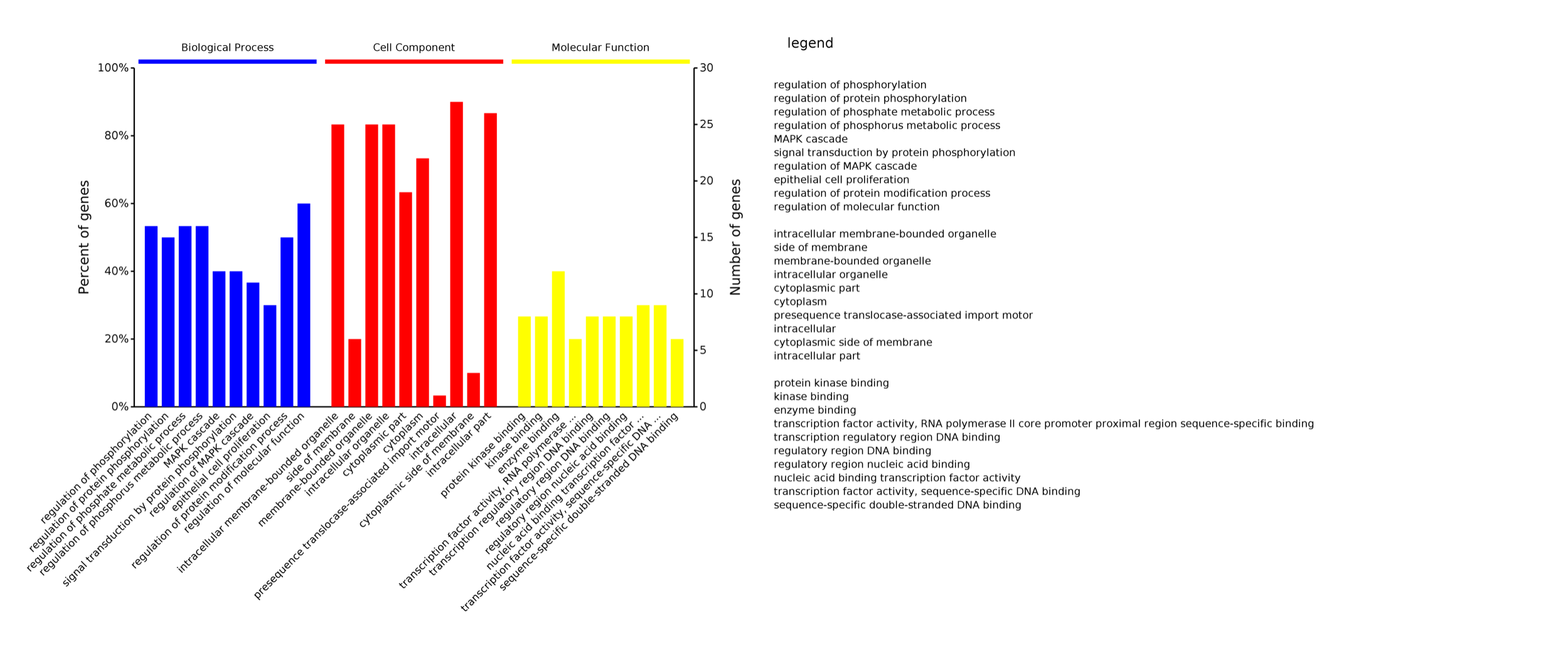
**

**Figure S3. GO enrichment of the 35 DEGs regulated by 10 μg/mL and 100 μg/mL PEP1 peptide**

## Supplementary Tables

**Table S1. Primers for reverse transcription PCR**

| **Gene** | | **Primer sequence (5’ - 3’)** |
| --- | --- | --- |
| ***Dusp2*** | Forward | TGGAAATCTTGCCCTACCTG |
|  | Reverse | CTCCTGGAACCAGGCACTTA |
| ***Fosl1*** | Forward | CGGAGACTGACAAACTGG |
|  | Reverse | CTCAGGTTCAAGCACAGG |
| ***Cd44*** | Forward | CCTCACATCCAACACCTCCC |
|  | Reverse | AGCAGTGGTGCCATTTCTGT |
| ***Thsb1*** | Forward | GAAAGACGCCTGCCCAATTAAT |
|  | Reverse | ACTTGATTTTCTGTCACATCGC |
| ***Dusp5*** | Forward | TCGCCTACAGACCAGCCTAT |
|  | Reverse | GTAGTGTAGGTGGGTGGTGC |
| ***Ier3*** | Forward | CAGCCGAAGGGTGCTCTAC |
|  | Reverse | AAATCTGGCAGAAGATGATGG |
| ***Stat6*** | Forward | TTCAACGACAACAGCCTCAG |
|  | Reverse | CTGGCTCATTGAGGAGAAGG |
| ***Traf4*** | Forward | CAGGAGAGTGTCTACTGTGAGA |
|  | Reverse | CCACACCACATTGGTTGGG |
| ***β-actin*** | Forward | GCCGGGACCTGACTGACTAC |
|  | Reverse | CGGATGTCCACGTCACACTT |

**Table S2. Summary of sample** **Post-Filter Polymerase reads**

| **Samples** | **Number of total subreads bases** | **Number of subreads** | **Mean subreads length** | **Subreads N50 length** | **Max Subreads length** |
| --- | --- | --- | --- | --- | --- |
| Control_subreads.bam | 58,332,789,094 | 25,497,942 | 2,288 | 2,949 | 228,986 |
| Pep10_subreads.bam | 58,863,538,838 | 25,510,744 | 2,307 | 2,877 | 192,604 |
| Pep100_subreads.bam | 68,413,566,530 | 27,850,613 | 2,456 | 2,964 | 195,687 |
| Total | 185,609,894,462 | 78,859,299 | 7,052 | 8,790 | 617,277 |
|  | | | | | |

**Table S3. Statistics of CDS in novel transcripts and novel genes**

| **CDS Type** | **Number** |
| --- | --- |
| confident-3partial | 195 |
| confident-5partial | 2989 |
| confident-complete | 1009 |
| confident-internal | 414 |
| dumb-3partial | 1781 |
| dumb-5partial | 3 |
| dumb-complete | 55849 |
| likely-NA | 7039 |
| suspicious-NA | 68744 |

**Table S4. Summary of annotation result**

| **Database** | **KOG** | **KEGG** | **NR** | **SwissProt** | **GO** | **Total** | **Overall annotated** |
| --- | --- | --- | --- | --- | --- | --- | --- |
| **Gene Number** | 93,928 | 69,205 | 127,482 | 119,762 | 80,278 | 138482 | 127,867 |
| **Annotation Ratio** | 67.83% | 49.97% | 92.06% | 86.48% | 57.97% | - | 92.33% |
|  | | | | | | |  |

**Table S5. Results of comparison with reference genome**

| **Sample name** | **Total reads** | **Total mapped** | **Multiple mapped** | **Uniquely mapped** |
| --- | --- | --- | --- | --- |
| **Control1** | 76497602 | 73248722 (95.75%) | 6615700 (8.65%) | 66633022 (87.10%) |
| **Control2** | 50787286 | 48584556 (95.66%) | 4330339 (8.53%) | 44254217 (87.14%) |
| **Control3** | 48356480 | 46381524 (95.92%) | 4394385 (9.09%) | 41987139 (86.83%) |
| **Pep10_1** | 56154042 | 53862256 (95.92%) | 5173018 (9.21%) | 48689238 (86.71%) |
| **Pep10_2** | 48705046 | 46609580 (95.70%) | 4176309 (8.57%) | 42433271 (87.12%) |
| **Pep10_3** | 61608096 | 59133360 (95.98%) | 5611333 (9.11%) | 53522027 (86.87%) |
| **Pep100_1** | 62874328 | 60238207 (95.81%) | 5491700 (8.73%) | 54746507 (87.07%) |
| **Pep100_2** | 55956404 | 53493972 (95.60%) | 4719315 (8.43%) | 48774657 (87.17%) |
| **Pep100_3** | 56106908 | 53659641 (95.64%) | 4894685 (8.72%) | 48764956 (86.91%) |

**Table S6. The expression levels of all genes**

| **Sample** | **0-0.1** | **Ratio** | **0.1-3.75** | **Ratio** | **3.75-15** | **Ratio** | **>15** | **Ratio** |
| --- | --- | --- | --- | --- | --- | --- | --- | --- |
| **Control1** | 24440 | 55.52% | 9630 | 21.87% | 5426 | 12.33% | 4527 | 10.28% |
| **Control2** | 24585 | 55.85% | 9402 | 21.36% | 5426 | 12.33% | 4610 | 10.47% |
| **Control3** | 24713 | 56.14% | 9369 | 21.28% | 5349 | 12.15% | 4592 | 10.43% |
| **Pep10_1** | 24712 | 56.13% | 9451 | 21.47% | 5263 | 11.96% | 4597 | 10.44% |
| **Pep10_2** | 24630 | 55.95% | 9371 | 21.29% | 5410 | 12.29% | 4612 | 10.48% |
| **Pep10_3** | 24416 | 55.46% | 9614 | 21.84% | 5434 | 12.34% | 4559 | 10.36% |
| **Pep100_1** | 24434 | 55.50% | 9579 | 21.76% | 5424 | 12.32% | 4586 | 10.42% |
| **Pep10_2** | 23992 | 54.50% | 9911 | 22.51% | 5525 | 12.55% | 4595 | 10.44% |
| **Pep10_3** | 24437 | 55.51% | 9556 | 21.71% | 5400 | 12.27% | 4630 | 10.52% |

Note: the data in the table are FPKM value distribution and expression statistics of biological repeat gene expression in different sample groups.

**Table S7. 139 DEGs including the FPKM, GO and KEGG (in a separate XLSX sheet)**

**Table S8. KEGG enrichment of genes involved in immune response**

| **KEGG pathway description** | **Gene count** | ***p*-value** | **Matching proteins in PPI network** |
| --- | --- | --- | --- |
| NF-kappa B signaling pathway | 10 | 0.043 | *Traf4, Tnf2, Tnf3, Tnf4, Nfkbiz, Gadd45g, Fosl1, Stat6, Fosl1, Egr1* |
| MAPK signaling pathway | 9 | 2.11E-05 | *Gadd45g, Ereg, Dusp2, Dusp5, Rasa1, Ier3, Tnf2, Tnf3, Tnf4* |
| TGF-beta signaling pathway | 9 | 6.34E-06 | *Tnf2, Tnf3, Tnf4, Thbs1, Bmpr2, Id1, Id3, Egr1, Ier3* |
| Epstein-Barr virus infection | 6 | 0.0021 | *Cd44, Gadd45g, Tnf2, Tnf3, Tnf4, H2-DMb2* |
| Human T-cell leukemia virus 1 infection | 6 | 0.0024 | *Tnf2, Tnf3, Tnf4, Fosl1, H2-DMb2, Egr1* |
| IL-17 signaling pathway | 5 | 0.0032 | *Traf4, Tnf2, Tnf3, Tnf4, Fosl1* |
| Inflammatory bowel disease | 5 | 0.0018 | *Tnf2, Tnf3, Tnf4, H2-DMb2, Stat6* |
| Proteoglycans in cancer | 5 | 0.0182 | *Cd44, Tnf2, Tnf3, Tnf4, Thbs1* |
| AGE-RAGE signaling pathway in diabetic complications | 4 | 0.0431 | *Tnf2, Tnf3, Tnf4, Egr1* |
| Allograft rejection | 4 | 0.0215 | *Tnf2, Tnf3, Tnf4, H2-DMb2* |
| Antigen processing and presentation | 4 | 0.0337 | *Tnf2, Tnf3, Tnf4, H2-DMb2* |
| Graft-versus-host disease | 4 | 0.0214 | *Tnf2, Tnf3, Tnf4, H2-DMb2* |
| Hematopoietic cell lineage | 4 | 0.00013 | *Cd44, Tfrc, Tnf, H2-DMb2* |
| Leishmaniasis | 4 | 0.0311 | *Tnf2, Tnf3, Tnf4, H2-DMb2* |
| Rheumatoid arthritis | 4 | 0.0427 | *Tnf2, Tnf3, Tnf4, H2-DMb2* |
| Systemic lupus erythematosus | 4 | 0.0427 | *Tnf2, Tnf3, Tnf4, H2-DMb2* |
| Toxoplasmosis | 4 | 0.044 | *Tnf2, Tnf3, Tnf4, H2-DMb2* |
| Type I diabetes mellitus | 4 | 0.0242 | *Tnf2, Tnf3, Tnf4, H2-DMb2* |
| MicroRNAs in cancer | 3 | 0.0113 | *Cd44, Thbs1, Bmpr2* |
| Phagosome | 3 | 0.0113 | *Tfrc, H2-DMb2,Thbs1* |
| Signaling pathways regulating pluripotency of stem cells | 3 | 0.0093 | *Id3, Bmpr2, Id1* |
| Colorectal cancer | 2 | 0.0427 | *Gadd45g, Ereg* |
| ECM-receptor interaction | 2 | 0.0427 | *Cd44, Thbs1* |
| p53 signaling pathway | 2 | 0.0328 | *Gadd45g, Thbs1* |
| Small cell lung cancer | 2 | 0.0427 | *Traf4, Gadd45g* |
| Th1 and Th2 cell differentiation | 2 | 0.0427 | *H2-DMb2, Stat6* |
| Th17 cell differentiation | 2 | 0.0431 | *H2-DMb2, Stat6* |
| JAK-STAT signaling pathway | 1 | 0.0032 | *Stat6* |

**Table S9. The interaction analysis via STRING**

| **Node1** | **Node2** | **Homology** | **Coexpression** | **Experimentally determined interaction** | **Database annotated** | **Automated textmining** | **Combined score** |
| --- | --- | --- | --- | --- | --- | --- | --- |
| Bmpr2 | Cd44 | 0 | 0 | 0.058 | 0.137 | 0.198 | 0.291 |
| Bmpr2 | Tnf | 0 | 0 | 0.047 | 0.132 | 0.337 | 0.403 |
| Bmpr2 | Fosl1 | 0 | 0 | 0.07 | 0.267 | 0.209 | 0.413 |
| Bmpr2 | Thbs1 | 0 | 0.062 | 0.045 | 0 | 0.299 | 0.317 |
| Bmpr2 | Stat6 | 0 | 0.062 | 0.136 | 0.122 | 0.132 | 0.3 |
| Bmpr2 | Id1 | 0 | 0.049 | 0 | 0 | 0.466 | 0.471 |
| Cd44 | Id3 | 0 | 0 | 0 | 0 | 0.25 | 0.25 |
| Cd44 | Ereg | 0 | 0.1 | 0 | 0 | 0.238 | 0.284 |
| Cd44 | Fosl1 | 0 | 0.113 | 0 | 0 | 0.331 | 0.381 |
| Cd44 | Egr1 | 0 | 0 | 0 | 0 | 0.399 | 0.398 |
| Cd44 | Stat6 | 0 | 0.094 | 0.066 | 0 | 0.386 | 0.435 |
| Cd44 | Thbs1 | 0 | 0.193 | 0 | 0 | 0.517 | 0.594 |
| Cd44 | Tfrc | 0 | 0 | 0 | 0 | 0.682 | 0.682 |
| Cd44 | Tnf | 0 | 0.104 | 0 | 0 | 0.743 | 0.76 |
| Dusp2 | Tnf | 0 | 0.319 | 0 | 0 | 0.215 | 0.442 |
| Dusp2 | Egr1 | 0 | 0.114 | 0.057 | 0 | 0.382 | 0.439 |
| Dusp2 | Nfkbiz | 0 | 0.27 | 0.078 | 0.108 | 0.24 | 0.482 |
| Dusp2 | Dusp5 | 0.909 | 0.144 | 0 | 0.499 | 0.757 | 0.583 |
| Dusp5 | Ier3 | 0 | 0.066 | 0 | 0 | 0.294 | 0.312 |
| Dusp5 | Tnf | 0 | 0.061 | 0 | 0 | 0.234 | 0.25 |
| Dusp5 | Fosl1 | 0 | 0.09 | 0.091 | 0 | 0.197 | 0.278 |
| Dusp5 | Ereg | 0 | 0 | 0 | 0 | 0.337 | 0.337 |
| Dusp5 | Thbs1 | 0 | 0 | 0 | 0 | 0.256 | 0.256 |
| Dusp5 | Nfkbiz | 0 | 0.061 | 0.078 | 0.108 | 0.168 | 0.271 |
| Dusp5 | Egr1 | 0 | 0.064 | 0.057 | 0 | 0.398 | 0.422 |
| Egr1 | Ier3 | 0 | 0.212 | 0 | 0 | 0.507 | 0.595 |
| Egr1 | Gadd45g | 0 | 0.062 | 0.045 | 0 | 0.284 | 0.302 |
| Egr1 | Tnf | 0 | 0.089 | 0.047 | 0.194 | 0.62 | 0.698 |
| Egr1 | Fosl1 | 0 | 0.096 | 0.058 | 0.13 | 0.639 | 0.697 |
| Egr1 | Thbs1 | 0 | 0.064 | 0 | 0 | 0.36 | 0.375 |
| Egr1 | Id1 | 0 | 0 | 0.058 | 0 | 0.309 | 0.321 |
| Egr1 | Stat6 | 0 | 0 | 0.078 | 0 | 0.294 | 0.321 |
| Egr1 | Nfkbiz | 0 | 0.385 | 0.085 | 0.114 | 0.35 | 0.632 |
| Ereg | Tnf | 0 | 0 | 0 | 0 | 0.396 | 0.396 |
| Ereg | Fosl1 | 0 | 0.163 | 0 | 0 | 0.22 | 0.319 |
| Ereg | Thbs1 | 0 | 0.127 | 0 | 0 | 0.26 | 0.326 |
| Fosl1 | Ier3 | 0 | 0.187 | 0 | 0 | 0.351 | 0.45 |
| Fosl1 | Traf4 | 0 | 0 | 0.25 | 0 | 0.113 | 0.306 |
| Fosl1 | Tnf | 0 | 0.094 | 0 | 0.337 | 0.404 | 0.61 |
| Fosl1 | Thbs1 | 0 | 0.121 | 0 | 0 | 0.216 | 0.281 |
| Fosl1 | Stat6 | 0 | 0.057 | 0.071 | 0.216 | 0.129 | 0.321 |
| Fosl1 | Nfkbiz | 0 | 0.136 | 0.058 | 0.12 | 0.179 | 0.334 |
| Id1 | Id3 | 0.837 | 0.423 | 0 | 0 | 0.763 | 0.494 |
| Id1 | Thbs1 | 0 | 0.069 | 0 | 0.8 | 0.319 | 0.862 |
| Ier3 | Tnf | 0 | 0.144 | 0 | 0 | 0.395 | 0.459 |
| Ier3 | Nfkbiz | 0 | 0.412 | 0 | 0 | 0.448 | 0.662 |
| Nfkbiz | Traf4 | 0 | 0 | 0.088 | 0.145 | 0.266 | 0.377 |
| Nfkbiz | Tnf | 0 | 0.421 | 0.058 | 0.24 | 0.499 | 0.764 |
| Nfkbiz | Stat6 | 0 | 0.074 | 0.079 | 0.151 | 0.197 | 0.341 |
| Rasa1 | Traf4 | 0 | 0 | 0.325 | 0 | 0 | 0.325 |
| Stat6 | Tnf | 0 | 0.069 | 0 | 0.35 | 0.703 | 0.804 |
| Tfrc | Tnf | 0 | 0 | 0 | 0 | 0.669 | 0.669 |
| Thbs1 | Tnf | 0 | 0 | 0 | 0 | 0.585 | 0.585 |
| Tnf | Traf4 | 0 | 0 | 0.215 | 0.334 | 0.473 | 0.701 |
